# Drug use and abuse in Europe: Mental health consequences

**DOI:** 10.1192/j.eurpsy.2025.72

**Published:** 2025-08-26

**Authors:** A. Schellekens

**Affiliations:** Psychiatry, Radboudumc, Nijmegen, Netherlands

## Abstract

**Abstract:**

Substance use is an onging public health burden. As a result all psychiatrists encounter patients with substance use and addictive disorders, either as primary mental health issue, or as co-occurring with other mental health issues. In this presentation, first trends in substance use across Europe will be presented, based on findings from the **European Union Drugs Agency (EUDA). Next, findings on co-occurring other mental health issues will be discussed, with a focus on co-occurring Attention Deficit Hyperactivy Disorder (ADHD). Recent findings from the International Colaboration on ADHD and Substance Abuse (ICASA) will be shared.** ICASA’s most recent work was the longitudinal International Naturalistic Cohort Study of ADHD and Substance Use Disorders (SUD). Nearly 600 patients with both ADHD and SUD in treatment were followed for nine months. About two thirds of participants received some kind of ADHD treatment, with half receiving pharmacologic treatment, one third receiving psychological treatment, and only one-fifth receiving combined pharmacologic and psychological treatment. Higher ADHD symptom severity and sobriety at intake were associated with receiving ADHD treatment. These findings suggest that treatment of SUD and ADHD is suboptimal. The implementation of ICASA’s consensus statements on diagnosis and treatment of co-occurring ADHD and SUD could help improve quality of care for these individuals.

**Disclosure of Interest:**

None Declared

